# Meaning-making and creativity in musical entrainment

**DOI:** 10.3389/fpsyg.2023.1326773

**Published:** 2024-01-03

**Authors:** Andrea Schiavio, Maria A. G. Witek, Jan Stupacher

**Affiliations:** ^1^School of Arts and Creative Technologies, University of York, York, United Kingdom; ^2^Centre for Systematic Musicology, University of Graz, Graz, Austria; ^3^Department of Music, School of Languages, Cultures, Art History and Music, University of Birmingham, Birmingham, United Kingdom; ^4^Center for Music in the Brain, Aarhus University and The Royal Academy of Music Aarhus/Aalborg, Aarhus, Denmark

**Keywords:** music, entrainment, creativity, meaning-making, coupling

## Abstract

In this paper we suggest that basic forms of musical entrainment may be considered as intrinsically creative, enabling further creative behaviors which may flourish at different levels and timescales. Rooted in an agent's capacity to form meaningful couplings with their sonic, social, and cultural environment, musical entrainment favors processes of adaptation and exploration, where innovative and functional aspects are cultivated via active, bodily experience. We explore these insights through a theoretical lens that integrates findings from enactive cognitive science and creative cognition research. We center our examination on the realms of groove experience and the communicative and emotional dimensions of music, aiming to present a novel preliminary perspective on musical entrainment, rooted in the fundamental concepts of meaning-making and creativity. To do so, we draw from a suite of approaches that place particular emphasis on the role of situated experience and review a range of recent empirical work on entrainment (in musical and non-musical settings), emphasizing the latter's biological and cognitive foundations. We conclude that musical entrainment may be regarded as a building block for different musical creativities that shape one's musical development, offering a concrete example for how this theory could be empirically tested in the future.

## Introduction

*Entrainment* is commonly defined as the process in which two or more biological or mechanical systems interact with each other, leading to various forms of temporal coordination (Clayton et al., 2005; Knoblich and Sebanz, 2008; Clayton, 2012). In music, as Trost et al. (2017) explain, the concept of entrainment has been adopted to describe the emergence and maintenance of different behavioral adaptations associated with the perception and production of temporal regularities and hierarchical structures in musical rhythm, such as breathing patterns, body swaying, hand clapping, and dancing. They also point out that in most contexts of music perception there is no reciprocal interaction between systems^1^; it is only the listener who adapts their movements to the auditory signal, while the latter remains substantially unaffected by the movements of the listener. However, experience tells us that temporal locking with an external musical stimulus may shape our engagement with the auditory signal as well as its meaning. This suggests a scenario in which fundamental dimensions of the auditory signal itself may undergo significant alterations. In other words, through entrainment, listeners have the capacity to reshape their connection with the music they encounter, thereby influencing not only their own experiences but also impacting the music itself. As such, musical entrainment might not primarily be conceived of as a response to a stimulus; rather it might be better understood as an active phenomenon in which meaningful aspects of the musical pattern one entrains to can be brought forth, allowing new and valuable experiences to be generated.

In this article, we combine the focus on such an understanding of musical entrainment with research on enactive and creative cognition, aiming to offer a new preliminary vision of musical entrainment that relies on the concepts of *meaning-making* and *creativity*. To do so, in the first part of the paper we draw from a suite of approaches that place particular emphasis on the role of situated experience as well as bodily action and explore a range of recent empirical work on entrainment (in musical and non-musical settings), emphasizing the latter's biological and cognitive foundations. In the second, and more speculative part of the paper, we examine if, and to what extent, entrainment phenomena may be seen to display a creative core. Here we propose that forms of musical entrainment observed in early childhood may be considered as intrinsically creative, as they appear to involve innovative and functional properties—two defining features of creativity (Runco and Jaeger, 2012). Because this may enable further creative behaviors which flourish later in life, musical entrainment might be considered, at least within specific developmental settings, as an ontogenetic precursor of creativity.

It is argued that such a perspective can help us look at entrainment from a new light, inspiring the development of novel hypotheses for future work in both musical and non-musical domains. With this in mind, it should be noted that our conceptual analysis primarily seeks to explore a theoretical landscape and its possible applications. As such, the present contribution cannot generate definitive answers or provide a complete taxonomy of the main (e.g., creative) properties of musical entrainment. Rather, our aim is to offer novel conceptual heuristics and resources that can inform future theoretical and experimental research into entrainment. With this in mind, our conclusive section includes a concrete example of how this theory could be empirically tested in the future.

## Entrainment and meaning-making

### Entrainment across different contexts

According to Clayton et al. (2005, p. 2–3), in all entrainment phenomena: (i) there are two or more *autonomous* oscillators or rhythmic processes that occur independently (e.g., one is not caused by the other), and (ii) there must be an *interaction* between these processes. The first point differentiates entrainment from mere resonance—where the exposure of one oscillating system to another reinforces the first oscillator's natural frequency amplitude. The second point implies that while not all interacting systems can entrain (it depends on whether the natural frequency of the systems involved are close enough to each other), all entrained systems are coupled, in the sense that the systems can affect each other. Entrainment can be found in various physical and biological systems, such as pendulum clocks, brains, and human sleep-wake cycles. For instance, temporally coupled systems may include neural networks (neural entrainment), body functions (physiological entrainment, such as in breathing), and body parts (motor entrainment, such as in dancing). All of these systems can be affected by music, and they can affect each other. In musical entrainment, the temporal regularity of the rhythm often constitutes one of the oscillating systems, which is coupled with another oscillating system in the listener (and the nature of these can vary). It is obvious that when listening to recorded music, the acoustic signal *per se* cannot be influenced by the listener; however, entrainment can affect the interpretation of the acoustic signal. For example, moving to the music in time with a specific rhythmic structure can influence meter perception, which London (2004) fittingly describes as “a musically particular form of entrainment” (p. 4).

On a social level, the intra-individual systems are not only coordinated with music, but also with other individuals or groups. This resonates with the notion of “musical social entrainment” recently developed by Kim et al. (2019), which refers to “intra-individual, inter-individual, intra-group, and inter-group entrainment to exogenous musical rhythms—including the rhythms of other musically acting individuals and groups—embedded in a social context and contributing to sociality” (Kim et al., 2019). While we recognize the analytical utility of the distinctions between specific levels of entrainment, we also observe that such categories are intrinsically connected to each other, combining neural, social, and motor dimensions at various degrees. As such—unless explicitly mentioned otherwise—we treat the word “entrainment” as an umbrella term that brings together these levels. This is in accordance with Trost et al.'s (2017) idea that “instead of separate independent processes, different levels of entrainment are components of the same phenomenon that can be measured in different ways” (p. 105). Consequently, “tapping into one level of entrainment triggers the others” (Trost et al., 2017).

In the brain, entrainment refers to “the temporal alignment of an observed neural process with the regularities in an exogenously occurring stimulus” (Obleser and Kayser, 2019, p. 913). Most neuroscientific studies use EEG or MEG to capture this temporal alignment of neural oscillations to external stimuli in form of so called steady-state evoked potentials (SSEPs). In the auditory domain, two EEG studies showed that SSEPs can occur with simple stimuli, such as isochronous pulse trains (Nozaradan et al., 2011), and with more complex stimuli, such as music (Tierney and Kraus, 2015). It was further shown that beat-locked neural oscillations can persist for a few seconds in silent periods after an external rhythm stops, suggesting that neural entrainment is at least partly a top-down controlled endogenous process and not pure resonance to the external signal (Stupacher et al., 2017b).

One of the first models to mathematically explore entrainment phenomena is the Haken-Bunz-Kelso coupled oscillator model (Haken et al., 1985), which was originally put forward to describe the spontaneous behavioral patterns emerging from various tasks involving bimanual coordination, such as synchronized finger-wagging. As Park and Turvey (2008, p. 4) report, this was developed from an early empirical study by Kelso (1984), which showed that there are two principal modes of coordination when moving our fingers rhythmically at a shared frequency: in-phase and anti-phase. Interestingly, in the same study, Kelso also observed the tendency for anti-phase patterns to spontaneously shift to in-phase during oscillations of higher speed, but not vice-versa. In other words, in-phase movement is a more stable state than anti-phase. These behavioral principles were later generalized to a greater variety of body-movements across different limbs, humans, and species of animals (Fuchs and Kelso, 2018; see also Tognoli et al., 2020). Furthermore, the anti-phase to in-phase switching at high speeds is mirrored in the phase relationships of neuronal ensembles in the brain, both within and between brains of interacting subjects (Kelso et al., 2013). Scholars working in this area were at first interested in gaining a richer understanding of the dynamics of human limbs involved in coordinated action, and eventually began to consider them as non-linearly coupled oscillators (Chemero, 2009). Coordinated behavior, on this view, unfolds through cycles of oscillatory processes that reciprocally influence each other in various ways, ensuring the overall coherence and (precarious) stability of sensorimotor action (Schmidt and Richardson, 2008).

In human beings,^2^ however, entrainment goes well-beyond tasks involving bimanual coordination. It serves a variety of functions and purposes—for example it “facilitate[s] complex and interdependent coordination that can be seen in human activities including sport and play, verbal communication and emotional expression, and in the epitome of rhythmic entrainment: music and dance” (Phillips-Silver et al., 2010, p. 3). And indeed, examples of motor entrainment involving humans are abundant in musical contexts. On a general level, the deep connection between music and movement has been recognized by a number of scholars (Mithen, 2005; Leman, 2007; Cox, 2016), and—as it will be discussed later in more detail—arguably plays a crucial role for musical meaning-making (Johnson, 1997; Clarke, 2005; Maes et al., 2014). More specifically, the systematic repetition of patterned behavior (i.e., executed by performers, audience members, etc.) is an important, if not defining, feature of joint musical activity (Merker et al., 2009).

As Levitin et al. (2018) report, there is ample literature on the emergence of coordinated behavior in response to music already in early infancy (e.g., Zentner and Eerola, 2010). A number of researchers have approached this phenomenon by exploring the patterns of interactions unfolding between caregiver and infant (Trevarthen, 1979; Trehub, 2003; Byrn and Hourigan, 2010), which includes vocal and gestural play. Here entrainment can be understood as a continuous mutual adaptation of different motor schema, which form and characterize such a coordinated interaction. A highly controlled study of entrained motor schema demonstrated that 14-month-old infants' helpfulness increases when moving to music in synchrony (Cirelli et al., 2014). Similarly, in adults, motor entrainment with others increases cooperation and affiliation (Hove and Risen, 2009; Wiltermuth and Heath, 2009) and music can strengthen these prosocial effects of motor entrainment (Stupacher et al., 2017a).

As mentioned earlier, entrainment can also occur in non-living systems (Rosenblum and Pikovsky, 2003). As reported by Cummins (2009), in the 17th Century the Dutch polymath Christiaan Huygens observed how two clock pendula tended to synchronize when placed on a common flexible surface:

“Recovering at home from an illness, he [Huygens] noticed that two pendulum clocks mounted in a single housing case inevitably generated non-independent pendular motions. Specifically, the two pendula consistently maintained an anti-phase relationship, whereby one pendulum would reach the mid-point of its cycle, just as the other was initiating its own cycle. The relationship was found to be insensitive to minor perturbation, such as introducing a transient delay in one pendulum. After perturbation, the pendula rapidly settled back into a regular anti-phase relation, or, as Huygens described it, an ‘odd sympathy”' (Cummins, 2009, p. 21).

Entrainment involves similar processes in both organic and inorganic systems. There is however one major difference between these occurrences, which gravitates around questions of meaning and experience. In biological systems—in humans, particularly—entrainment is *meaningful*; whether with a stimulus or with other persons. It can bring forth a vast array of feelings, ideas, and impressions about oneself and other(s); it can shape behavior, action plans, and ongoing social interactions in a rich variety of ways; and it may lead to novel emotional and behavioral configurations that can potentially affect how we perceive the world around us, including music. How can we account for such a characterization of entrainment, so intimately associated with meaning-making? While recent research has offered novel ways to capture important aspects of musical entrainment from a range of perspectives (Clayton et al., 2005; Trost et al., 2017), we arguably still lack adequate terminology and scientific resources to address its significance and values in human terms (Cummins, 2019). In what follows, we begin to explore how musical entrainment affords meaning, by approaching it as a particular case of coupling.

### From coupling to meaning-making

Entrainment is a form of coupling. In physics, coupling generally means exchange of information between two or more interacting objects or systems. In classical mechanics, coupling is often exemplified by a spring connecting two moving pendula, and the coupling strength is determined by the force of the spring. By definition, the force of the spring is distributed, because it is literally placed between and connects the two pendula; as such, the force is transferred back and forth through continuous feedback loops. In the case of two pendula standing on a common support (the Huygens' scenario), the coupling is partly determined by the mass and flexibility of the common support, that is, its aptness to conduct and transfer the force between the two pendula.

In a way similar to that of entrainment, the broader notion of coupling is often adopted to explain a variety of phenomena across the physical, biological, and cognitive sciences. The term plays a key role in the sciences of mind, especially in the approach known as *enactive cognitive science* (Varela et al., 1991; Thompson, 2007; Stewart et al., 2010; Di Paolo et al., 2017; Gallagher, 2017). This latter orientation conceives of mental life as a form of relational adaptiveness vis-à-vis an evolving ecology and has been recently applied to music research in a rich variety of ways (e.g., Kozak, 2019; Reybrouck, 2020; van der Schyff et al., 2022). Within this approach, organisms are at the same time conceived of as *biologically autonomous* and *coupled with their environment*. Enactivist theory, in other words, proposes that living beings are both self-directed and inseparable from their surroundings: organisms can regulate their own internal processes, adapt to changing conditions, and make choices based on their own needs and goals—which are also determined by the ecological niche they inhabit and shape. When considering biological autonomy…

“… the basic idea is that living beings generate and maintain themselves. Stated more abstractly, an autonomous system is a self-generating and self-sustaining system. The theory of autonomous systems takes living systems as the paradigm and focuses on explaining the emergence and constitution of individuality, agency, and functional and behavioral norms” (Thompson, 2018).

As said, while being autonomous, organisms are also coupled with the environment (Di Paolo, 2005; Torrance and Froese, 2011; De Jesus, 2015). Thompson (2007) lays out three main criteria for enactive coupling of an agent with the environment: (i) the process is dynamic such that the interacting systems become co-dependent (where changes in the systems depend mutually on each other), (ii) the mutual interaction results in a coherent supra-system, (iii) and the agent retains its autonomy despite its co-dependence on the other system (see also Varela, 1979). These criteria are important for casting coupling as a constitutive property of cognitive systems, rather than as a result of a causal relation (e.g., from a posited central executive, such as the brain). Living systems, accordingly, are not conceived of as “things among other things”—objective, measurable, constituents of an a-priori environment—but are rather seen as inherently relational, situated, and animated beings who survive through viable forms of organism-environment interactivity (De Jaegher and Di Paolo, 2007; Kyselo, 2014; Villalobos and Ward, 2015; Rojas-Líbano and Parada, 2020). Within this framework, the notion of coupling is adopted to explain how mental life might be seen to be distributed across organisms and agents, at cellular, organismic, corporeal, personal, and social levels (Maturana and Varela, 1980; Thompson, 2005; Froese and Di Paolo, 2011). By this view, coupling permeates our life in various ways, enabling situated experience and abstract thought: we cannot think about, or experience, things without a functioning brain-body system; and we cannot have a functioning brain-body system without a niche in which the brain-body system is situated and with which it is coupled.

Enactivists argue that mental life might be best understood as a relational activity brought forth by motivated patterns of action and interaction, where significance and values associated with our kinematic experiences permit the preservation and flourishing of our identity (Thompson, 2007; Barandiaran, 2017; De Jesus, 2018; Gallagher, 2020). This point helps us better understand how biological autonomy and coupling intersect. Consider the definition of cognition offered by Di Paolo: “[c]ognition is sense-making in interaction: the regulation of coupling with respect to norms established by the self-constituted identity that gives rise to such regulation in order to conserve itself” (Di Paolo, 2009, p. 19). Entrainment may be thus conceived of as a particular form of coupling that enables living systems to interact with, and actively shape, the environment in which they are situated. Motor activities and experiences associated with entrainment may thus become meaningful in the sense that they disclose an open horizon of possible interactive configuration that would sustain and enrich the organism-world coupling in various ways (Chemero, 2009; Sheets-Johnstone, 2015; Di Paolo et al., 2017).

This may involve sophisticated movements generated in practices such as dance, sport, and music performance, as well as much simpler, spontaneous motor activities that emerge in infancy (Schiavio et al., 2017; Kozak, 2019; Høffding and Schiavio, 2021). Consider the common phenomenon of music-induced foot-tapping: it is well-documented that humans can entrain their body-movements both consciously and subconsciously to a beat (Stephan, 2002). Such natural, self-sustained movement can produce important differences in how we attend to the musical stimulus (with or without awareness). It provides novel ways to *explore* the environment and engage with it through spatiotemporal coordination. We say “explore” because by entraining with music, novel motor configurations may emerge, allowing new possible movements (e.g., dance) to be discovered and developed. In turn, this may also increase one's understanding about music (e.g., in case of polyrhythms^3^ one may entrain to different pulses, thus experiencing a variety of musical-temporal features), and about the self (e.g., the capacity to actively switch from one rhythm to another can be re-adapted to different contexts, leading to richer experiences and novel behaviors).

**Figure 1:**
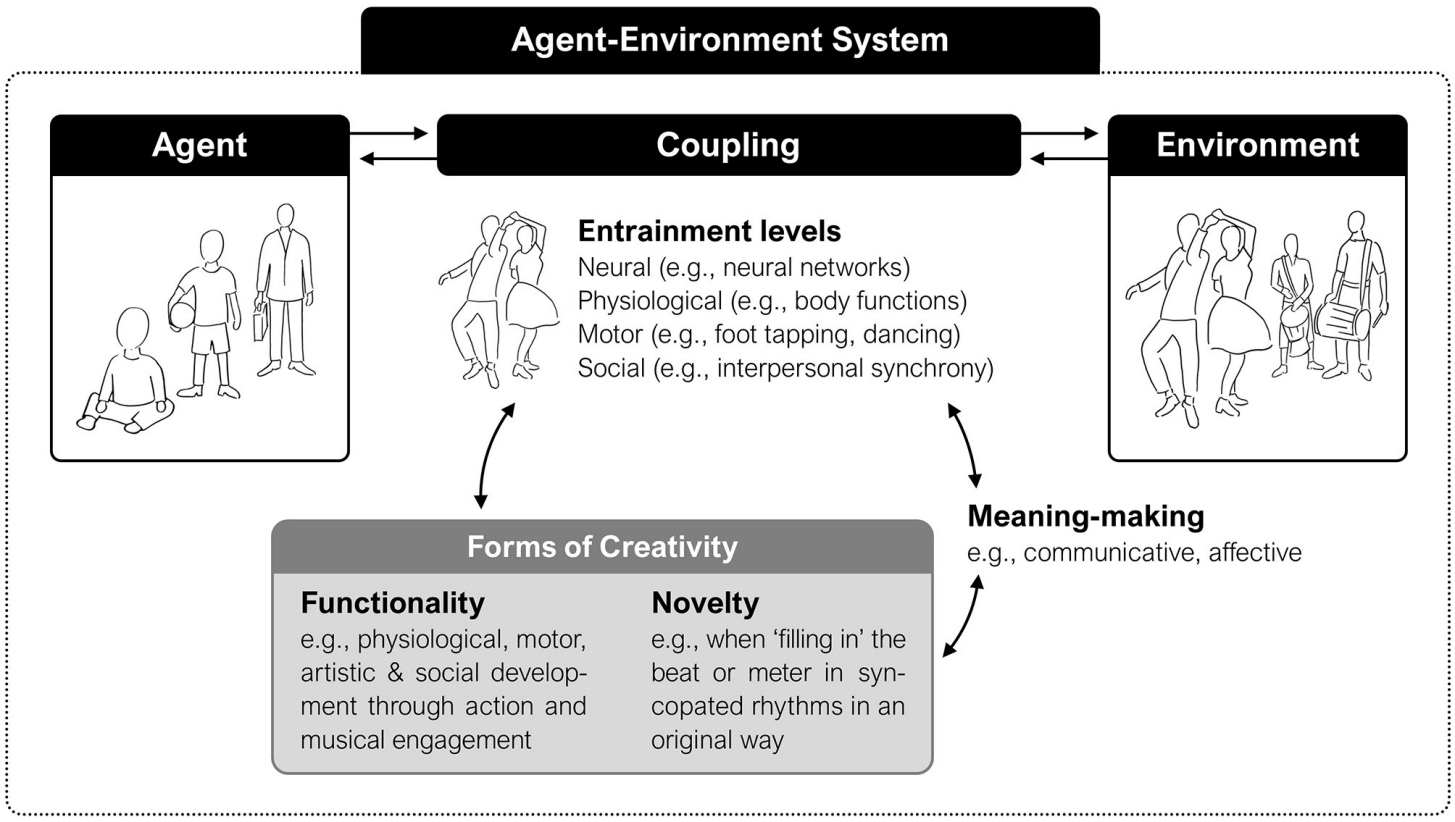
Entrainment may be conceptualized as a creative capacity to establish, maintain, and renew a meaningful perspective over a sonic and social ecology via bodily-based forms of coupling.

Such layers of meaning-making can also play a role in more complex relational activities, inviting different forms of interaction to be developed. Indeed, entrainment (as a human phenomenon) may be regarded as an active way to engage with the environment—one which fosters the generation of novel social and embodied experiences (Figure 1). Its emergent dynamics might therefore offer the agent an opportunity to negotiate and generate meaning across different layers of bodily awareness and social relations, instantiating organism-world couplings at various levels and timescales. It has been recently argued that for such couplings to be meaningful, two defining properties of creativity (i.e., *functionality* and *novelty*) are essential (Schiavio and Benedek, 2020). As we will see, such properties might be best observed in particular instantiations of musical entrainment unfolding in infancy. This insight prompt us to reflect on the possible role that entrainment might play for the development of creative thinking and behavior, helping us contribute a novel perspective on this phenomenon. Before exploring this dimension in more detail later, we first provide examples from empirical work examining the relationship between entrainment and meaning in multiple ways.

### Affective entrainment and groove

Emotion is perhaps one of the most obvious forms of meaning that musical entrainment can afford. Entraining your attention to the beat of a “groovy” rhythm (Danielsen, 2006) or your body-movements to the dance moves of a crowd in a club can elicit intense emotional and pleasurable experiences (Malbon, 2002; Witek, 2019). Nonetheless, the relationship between affect and entrainment has only recently been theorized (Vuilleumier and Trost, 2015; Trost et al., 2017) and explored empirically. In a recent study, Labbé and Grandjean (2014) asked participants to listen to different recordings of a classical music piece for solo violin and rate their experienced emotions (as recorded on the Geneva Emotion in Music Scale, GEMS, see Zentner et al., 2008), overall affect, and felt entrainment. The authors found that both visceral (e.g., feelings of change in internal rhythms) and motor entrainment (feelings of wanting to move a part of the body in time with the music) predicted a number of emotions, including joy, transcendence, wonder, power, and tenderness, as well as overall affective experience. Using more implicit measures of emotion based on ratings of emotional faces (which is known to be biased by participants' emotional states, see Niedenthal et al., 2000), Rabinowitch and Cross (2019) extended these findings to children aged 9–15 with no musical training. They found that tapping a rhythmic pattern in time with an experimenter produced more positive ratings of emotional faces than tapping asynchronously, suggesting that the children's emotional state is more positive during entrained musical behavior. There is also some neuroimaging evidence to support these behavioral findings. Using functional magnetic resonance imaging (fMRI), both Kokal et al. (2011), as well as Trost et al. (2014) found that the brain's caudate nucleus, which is associated with reward processing (Schultz, 2016), shows increased activation during sensorimotor and attentional entrainment. In fact, the caudate is part of both reward and motor networks in the brain (Lehéricy et al., 2006). Considering that emotion has been reported as the most important reason why people listen to music (Randall and Rickard, 2017), this research suggests that entrainment is one possible route to musical *eudaimonia*, i.e., musical-affective experiences embedded in meaningful values and purposeful engagement (Stark et al., 2018).

There is a special type of affective experience with music that is closely related to entrainment: groove. The repetitive movement of crowds dancing to the pulsating beats played by DJs in dance clubs is perhaps the most overt manifestation of pleasure in music. Psychologically, groove is defined as a pleasurable desire to move the body in time to the pulse of music (Madison, 2006; Janata et al., 2012; Senn et al., 2019). There is neurophysiological evidence showing that the sensation of groove modulates motor cortex excitability (Stupacher et al., 2013) and correlates with neural entrainment, measured as inter-phase EEG coherence (Cameron et al., 2019). Furthermore, the sensation of groove is associated with the fMRI-recorded activity of reward-related areas of the brain, including the nucleus accumbens, orbitofrontal cortex and caudate (Matthews et al., 2020).

Related to this affective significance is the communicative meaning of groove and entrainment. Doffman (2009) interviewed a jazz trio about their experiences and understandings of groove—here, defined as a feeling of temporal engagement between players that has both motional and social-communicative qualities. Entrainment was also measured quantitatively through onset detection algorithms applied to a recording of the trio's performance. These analyses illustrate not only *that* the trio entrains, but also *how*, that is, the characteristic micro-temporal relationships between the players defined by Keil (1995) as “participatory discrepancies”. Keil assumes that the temporal imperfections between players that generate an “in sync but out of phase” feeling are negotiated almost unconsciously and based on tacit knowing. In Doffman's interviews, the players described these entrainment qualities explicitly as communicating the various *feelings* that the musicians were attempting to express, and groove was seen to offer different sets of communicative possibilities. In a similar study, Walton et al. (2018) used systems dynamics models (similar to the Haken-Bunz-Kelso model described above) to evaluate differences in movement coordination and playing behavior in improvising piano duos and recorded the players' subjective experiences of playing. The modeling revealed coordinated turn-taking dynamics in both the acoustic and socio-behavioral patterns of interaction of the duos, and as in Doffman's study, different coordination regimes were described as producing opportunities to co-create different musical qualities, leading to individual creative freedoms within socio-musical exchanges. In this way, entrainment is not just a precondition for communication, but is instead carrying a “network of communicative dimensions” (Doffman, 2009, p. 144).

This is of course also true of linguistic communication. Evidence of entrainment has been shown in a number of properties of spoken language, including speaking rate (Giles et al., 1991), pitch (Levitan et al., 2012), intensity (Local, 2007), as well as changes in body posture, gesture, and facial expressions (Louwerse et al., 2012). The functional significance of entrainment in spoken language has been attributed to various mechanisms, including prediction (Zoefel, 2018) and memory (Albouy et al., 2017), which are all critical in meaning-making and information exchange. Behavioral entrainment during conversation is further known to contribute to rapport and empathy between conversing subjects (Miles et al., 2009). Therefore, “entrainment […] serves as a powerful coordinating device, uniting individuals in time and space to optimize comprehension, establish social presence, and create positive and satisfying relationships” (Borrie and Liss, 2014, p. 816).

These effects of entrainment on social attitudes and relationships have also received significant attention in music psychology research, with effects found on affiliation (Hove and Risen, 2009), cooperation (Kirschner and Tomasello, 2009), helpfulness (Cirelli et al., 2014), liking (Launay et al., 2014) and empathy (Carlson et al., 2019). Stupacher et al. (2017a) found that when watching videos of animated figures walking—where one figure represented the participant and the other another unknown person—participants' liking toward the other figure increased when the two figures were walking in synchrony with each other and with music, but not with a metronome. In another study, involving finger-tapping to synchronous or asynchronous music and metronome, participants displayed increased helpfulness toward a synchronized partner when tapping with music, but there were no effects of synchrony when tapping with a metronome and no effects on self-report measures of liking (Stupacher et al., 2017c). This difference of effects on implicit measures of prosocial behavior and self-reports suggests that entrainment can transform social relationships between people even in the absence of conscious awareness. Such socio-affective transformations, we propose, are enabled by particular creative properties that can be found at the core of musical entrainment and are particularly visible when looking at basic forms of entrainment in early infancy. In what follows, we thus extend our analysis to explore what we define as the “creative impulse” situated at the roots of entrainment and address its capacity to give rise to a range of creative outcomes associated with musical exploration, action, affect regulation, and interaction.

An example of the development of such new perspectives is the emotional and communicative meaning of groove. Several studies have shown that rhythms with a moderate amount of syncopation—i.e., notes that occur on weak metric positions followed by pauses on strong metric positions—elicit the strongest pleasurable desire to move (Sioros et al., 2014; Witek et al., 2014; Matthews et al., 2019; Stupacher et al., 2022b). Theoretically, it may be surprising that such metrical irregularity will promote synchronized body movement, as we see with groove (Witek et al., 2017). However, the conundrum becomes less puzzling if we consider that moderately syncopated beats offer opportunities for listeners and dancers to *fill in* the beat with their body movements. As Witek (2017) has proposed, syncopation opens up gaps in the music's rhythmic surface, which listeners and dancers are invited to actively move into by synchronizing their movements to the underlying beat. In this way, moderate levels of syncopation require listeners to *enact* the beat itself, echoing insights from theories of enactive cognition (Varela et al., 1991; Thompson, 2007; Di Paolo et al., 2017).

Remarkably, enacting the beat structure when entraining to syncopated music is productive in the sense that listeners and dancers are literally creating music (understood not just as auditory information but as an embodied form of agent-world interaction) with their movements. Without filling in the gaps—whether overtly through dance or covertly through attentive listening—the music would not be complete. The meaning formation of groove is thus closely tied to the creative properties that entraining to moderately syncopated beats can afford. The open spaces (or structural gaps) engendered by syncopation may be thus considered as venues through which listeners and dancers can on the one hand explore their creative impulses and express their individuality, while the collective expression of the underlying beat on the other hand can facilitate affiliation (Stupacher et al., 2022a, 2023). The importance of syncopation in social interactions with music was highlighted in a recent study by Stupacher et al. (2020). The authors found that social bonding with a virtual other person tends to follow an inverted *U*-shape with higher ratings of social closeness when moving together to rhythms with a moderate compared to low or high level of syncopation. A certain amount of complexity also seems to be relevant for affective responses in movement interactions without music: When playing the Mirror Game (i.e., two people freely move their hands as coordinated as possible), pairs who were more synchronized and produced more complex movements liked each other more (Ravreby et al., 2022). Both last-mentioned studies suggest that although simpler patterns make it easier to perceive and produce synchronized movements, a certain amount of complexity and novelty may promote affective responses. The choreographic ways in which one might resolve moderately complex rhythmic patterns and move into empty spaces created by syncopations will of course depend on one's cultural and historical situatedness, reflecting the development of different rhythm and movement patterns across space and time. On the dance floor, these venues are co-inhabited by multiple, interconnected agents, enabling the sometimes intensely transformative and socio-creative meanings that dancing in a group can afford.

## Entrainment as a potential building block of creativity?

Musical entrainment, as we saw, can bring about a rich domain of meaning in which one's engagement with the world is potentially transformed and (re)negotiated through motivated interaction with other processes (e.g., a musical pattern, a person) in our environments. And indeed, the empirical work we surveyed in the last section points to the key role of emotion and social dynamics in this coupling process, in both musical as well as non-musical contexts. A focus on meaning-making through interaction is also central to recent research on creativity (e.g., Montuori and Purser, 1995, 1999; Glãveanu, 2014) and musical creativity (e.g., Cook, 2018; Odena, 2018; Schiavio et al., 2022c). In this section, we present insights concerning the bio-cognitive nature of entrainment and its potential role as ontogenetic enabler of creativity. We examine such a conjecture in light of the previously introduced scholarship in enactive cognitive science and of recent work by Schiavio and Benedek (2020). In the latter, it was argued that a living system's capacity to act, survive, and flourish may depend on its ability to instantiate *novel* yet *functional* adaptive couplings with its environment. This establishes a striking similarity between mental life in general and creative processes in particular, when both…

“…can be conceived of as […] process[es] whereby agents actively shape and at the same time adapt to the environment in which they are situated. This […] gives rise to open-ended adjustments in thought and action, allowing agents to creatively (re-)establish, assemble, and decompose different organism–world relationships. We say *creatively*, because these relationships exhibit two properties – *novelty* and *functionality* – that are defining of creative activity and that many scholars would deem creative. Indeed, for such relationships to be “successful,” they must continuously renew themselves without moving too far from the contextual landscape from which they originate” (Schiavio and Benedek, 2020).

As we have suggested, musical entrainment may not only be understood as an automatic response-mechanism to a given stimulus; it may also be conceptualized as a natural, dynamic capacity to establish, maintain, and renew a particular perspective over a sonic and social ecology via bodily-based forms of coupling. When looking at infants, as we shall see, entrainment phenomena often display innovative and functional features. So, while we may not regard the entire phenomenon of musical entrainment in its variety of manifestations as inherently creative on its own, such an overlap of properties occurring in a specific time-window might make of entrainment a potentially central mechanism for the ontogenetic development of creativity.^4^ In what follows, we substantiate this claim by looking at early-life musical behaviors and trace a continuum between musical entrainment and creativity.^5^ Both entrainment and creativity seem to depend on one's ability to generate meaning via reciprocal organism-world interactions. Therefore, we should examine how the main properties of said couplings overlap or differ between both domains, to gain richer insights into if and how entrainment and creativity are related. Of particular interest for this analysis are forms of musical entrainment that emerge and develop early in life (Drake et al., 2000; Zentner and Eerola, 2010; Cirelli et al., 2016; Ilari et al., 2018), which we compare with the first musical “conducts” that often permeate the infant's musical and creative flourishing (Delalande, 2009, 2013).

Importantly, the capacity to entrain with music is not fully developed at birth. Ilari (2015) notes that infants cannot accurately synchronize with music at an early age (Provasi and Bobin-Bègue, 2003); and indeed, musical entrainment “takes time to fully mature and may be affected by several other factors, including types of tasks and specific demands (i.e., tapping, vocalizing, marching), stimuli tempi (Rose et al., 2012), music training (Drake et al., 2000), culture (Kirschner and Ilari, 2014), and social context (Rainbow, 1981; Schleuter and Schleuter, 1985; Eerola et al., 2006), to name a few” (Ilari, 2015, p. 334). This points to an understanding of musical entrainment as a developmental tendency associated to a meaningful engagement with music, which flourishes over time. By this view, early forms of entrainment may be seen to provide a privileged first way for infants to actively gain access to the world they inhabit, paving the path to more deliberate creative processes involved in the manipulation and development of the various sonic patterns and social interactions that characterize more sophisticated forms of entrainment (e.g., dancing to a rhythmical pulse, clapping hands on and off the beat, etc.). At the same time, these early forms of musical entrainment may be considered as minimally creative on their own, because they allow the infant to develop an *innovative* perspective over their sonic ecology, *functional* to their sensorimotor development (i.e., the ability to control movements, to associate sounds and actions, etc.), as well as to their own musical flourishing. As such, early forms of musical entrainment can be conceived of as minimally creative phenomena that may be regarded as a building block of different musical creativities that are developed later in life.

We refer here to the natural inclination of infants to generate sensorimotor relationships with their surrounding environment, thereby acquiring basic musical skills in the process (Delalande and Cornara, 2010; Schiavio et al., 2017; Peñalba et al., 2021). As infants explore and develop the motor skills necessary to manipulate objects, their movements can reflect early forms of music-like behaviors (Trevarthen, 1999): they can grab objects that are particularly affording of a given musical action (e.g., a squeezable toy that produces a funny sound), improving at the same time motility and perceptual abilities, including the capacity to associate sounds to movements. Infants do not develop these skills in a vacuum; there are important social contingencies involved in their own personal and artistic growth. Caregivers, for instance, facilitate their interactions with sounds and gestures; they reinforce the utterances made by the infant by mimicry and paraphrase; and they stimulate and motivate the infant to develop novel exploratory behaviors (e.g., Gratier and Trevarthen, 2008; Mazokopaki and Kugiumutzakis, 2009). Creativity is integrally involved here, as infants often explore different vocalizations, typically developing novel variations on existing patterns (Papoušek and Papoušek, 1981; Murray and Trevarthen, 1986; Bjørkvold, 1992; Benetti and Costa-Giomi, 2020). In such contexts, creativity unfolds through the various relationships that infants discover with their caregivers and develops in the context of their physical and social environment (see also Koops, 2014; Costa-Giomi and Sun, 2017).

We suggest that such creative endeavors may have roots in early forms of musical entrainment, providing the infant with a first *active*, bodily experience of musical engagement. In this sense, we can trace a continuum between the earliest forms of synchronization with music and the natural tendency that infants display to explore the world via action and interaction—a tendency that often characterizes their early musical development. In other words, it could be argued that preliminary body-world couplings established via musical entrainment are further developed and transformed into other forms of creative behaviors involving more complex patterns of sensorimotor, exploratory activity. Certain forms of entrainment and exploratory activities motivated by music give rise to organism-environment synergies that are both, albeit at different degrees, *innovative* and *functional*.

To further unpack this idea, let us compare two scenarios. The first involves an infant (name them Alex) entraining motorically to a musical pattern; the second involves another child (name them Charlie) exploring their surrounding environment through musical play. In the first case, following Zentner and Eerola (2010), we could imagine Alex being significantly more excited to move to a musical pattern when compared to speech, exhibiting enough motor flexibility to engage fluidly with the different rhythmic nuances of the auditory stimulus. We may observe the infant “accompanying” the music with the whole body. And as Alex rhythmically coordinates to the music, perhaps while sitting on the lap of a caregiver, *novel* experiences and sensorimotor associations could be discovered and familiarized with through movement. Among others, the experience of feeling closer to or further away from the sound source or the caregiver might be particularly *functional* to sustain the body-world relationships that such forms of entrainment often entail.^6^ Such experiences, in other words, appear to bring forth a specific domain of meaning framed relative to their innovative and functional (i.e., creative) properties. Compare now Alex with Charlie, who playfully explores their physical environment by actively producing music-like behaviors (e.g., by rhythmically squeezing a toy). Following the theoretical analysis put forward by Schiavio et al. (2017), the innovative and functional properties of such behaviors may be found in how Charlie manipulates the toy by employing specific motor sequences which, in turn, (i) disclose novel understandings of the (sonic) properties of the object and (ii) reveal richer opportunities for action by reinforcing existing motor configurations. Said differently, the sets of actions Charlie can employ in such music-exploratory activities may serve a double purpose when they improve their comprehension of the object (and its musical-affording properties), and help the infant develop new repertoires of action through further engagement in exploration.

The scenarios involving Alex and Charlie we have just described may help us consider how some forms of early childhood musical entertainment and exploratory musical play can both be viewed as creative, but in slightly different terms (as their overt behaviors is different). Such overlaps, while limited, point to a potentially shared developmental origin of creativity and entrainment. Musical entrainment in infancy is often innovative and functional, but the “innovative” aspects at the core of said behavior may soon vanish when the infant keeps on experiencing entrainment and gains enough familiarity with the possibilities that moving rhythmically to a musical stimulus offers. As such, entrainment may become less “original,” “unique,” or “unusual,” in one's development. This, we suggest, reinforces the natural drive of infants to explore their world further, actively looking for less familiar stimuli which may foster musical and creative flourishing.

As such, musical entrainment might be understood as a precursor of creativity, playing a potentially pivotal role in the latter's ontogenetic development. At first, the idea may seem counterintuitive when considering how creative faculties have normally been associated with rational thought, planning, and exploration of concepts (Boden, 1998). However, researchers working in areas such as that of ecological dynamics (e.g., Araújo et al., 2006) and musical creativity (e.g., van der Schyff et al., 2018) have long discussed how creative action—for instance in the contexts of sport and the performing arts—may be connected with real-time dynamics emerging within the moment-to-moment engagement between organisms and the physical and social environment in which they operate (Hristovski et al., 2012; Araújo et al., 2017; Kimmel, 2017, 2019; Schiavio and Kimmel, 2021). It has been argued that agents are often able to develop creative strategies and motor configuration to adapt to the online constraints of their activity by arranging, transforming, and re-deploying extant motor patterns on-the-fly (see also Gesbert and Durny, 2017; Gesbert et al., 2017; Orth et al., 2017; Schiavio et al., 2022a,b). Such an action-based understanding of creativity shifts the focus away from planned behavior and reasoning, toward the visceral, motor, conative, and interactive factors that permeate intentional action and its role in establishing dynamical relationships between organism and the world.

A possible avenue for investigating the intricate relationship between musical entrainment and the ontogenetic development of creative capacities involves longitudinal experimental designs. The main objective of such experiments is to ascertain whether distinct modes of exposure to musical entrainment significantly influence the trajectory of creative cognitive maturation. In pursuit of this endeavor, a heterogeneous cohort of participants, comprising children and adolescents from diverse cultural backgrounds, could be recruited and split into two groups. This methodological approach holds the potential to capture the nuanced variations in the impact of musical entrainment, encompassing a rich spectrum of meanings and actions associated with this phenomenon. Over the course of several weeks, the first group would partake in activities crafted to foster musical entrainment. These activities may encompass rhythm-centric improvisational exercises, dance routines, and the act of synchronously tapping to a musical stimulus. The second group would engage in non-musical and unsynchronized group activities. At predefined intervals, participants' creative abilities would be subjected to assessment using traditional selected creativity evaluation tools, looking at both divergent and convergent thinking abilities. Assessments of divergent thinking typically employ quantitative and qualitative scoring methods. Quantitative evaluation involves quantifying the total number of responses (referred to as ideational fluency) or the number of responses spanning diverse categories (known as ideational flexibility) generated within a specified time frame. In contrast, creative quality is frequently appraised through ratings provided by evaluators, comparison with established norms, or scrutiny for statistical rarity (Barbot et al., 2019). Convergent thinking tasks, on the other hand, focus on assessing creative problem-solving abilities. Notable examples include the Remote Associates Test—aka RAT (Mednick, 1968)—which requires identifying a word that connects three unrelated terms, and insight problems such as the nine-dot problem (Gilhooly and Murphy, 2005). Lazonder et al. (2022) have noted that while the reliability and validity of the RAT have been well-established with adult participants, its applicability to children has been explored to a limited extent. An exception to this is the study conducted by Howe et al. (2011), which demonstrated that children performed relatively well on particularly easy RAT problems (see Bowden and Jung-Beeman, 2003). The ability of children to tackle RAT problems is further underscored by Lazonder et al. (2022), who developed a test called RATje and evaluated its psychometric properties in two samples of Dutch upper-elementary school children. The results indicate that RATje offers a reasonably reliable and valid assessment of children's convergent thinking skills. In addition to divergent and convergent thinking assessments, more complex creative production tasks could be employed. These tasks involve creating drawings, composing narratives, or engaging in musical improvisation. Evaluation of performances in these tasks typically involves the use of expert panels (a technique known as consensual assessment; see Amabile, 1982). The analytical phase of this inquiry would be characterized by a scrutiny of the fluctuations in creative abilities occurring within each group and the disparities that manifest between them over time. We would expect that the group engaging in musical entrainment tasks exhibits a significantly pronounced rate of advancement in creative capabilities. This would yield preliminary corroboration of the thesis that musical entrainment indeed assumes a central role in the ontogenetic evolution of creativity.

## Conclusions

In this paper we have suggested that basic forms of musical entrainment may be considered as intrinsically creative, enabling further creative behaviors which may flourish later in life. Rooted in an agent's capacity to form meaningful couplings with their environment, musical entrainment favors processes of adaptation and continuous exploration,^7^ where innovative and functional aspects are cultivated and nurtured via active, bodily experience. Agents may develop novel perspectives over sounds and action, forming a concerned identity, or point of view (Thompson, 2007; Schiavio and van der Schyff, 2018) that is conducive to musical growth and, as such, functional to artistic and sensorimotor discovery.

So, while creativity is often understood as the capacity to develop something innovative and useful (Runco and Jaeger, 2012), it arguably remains based in the ability to establish organism-world couplings that, for music, begin with entrainment (Schiavio and Benedek, 2020). We have discussed how music and movement give rise to meaningful experiences that shape the various relationships formed between living systems and their musical niche through the lenses of emerging enactive scholarship (e.g., Reybrouck, 2020). In doing so, we have highlighted how mental life involves the constitution of sensorimotor, adaptive relationalities, and how these develop across ranges of actions-as-perception, sedimented in a history of structural couplings between organisms and their world.

The continuous co-specifications between living agents and their ecology brought forth in the process are crystallized in the development of a concerned perspective, or identity, which agents develop to (re)establish, sustain, stabilize, or optimize, said organism-world relationships. This point led us to consider how creative cognition may also be based in a similar organism-world circularity, as exemplified in musical discoveries and entrainment processes in early life, as well as in groove. Musical entrainment and creativity variously shape how living systems and their musical environment relate with each other. In both cases meaning can be transformed, and new motivated patterns of action can be developed and redeployed in various musical contexts. By this view, early forms of musical entrainment in infancy may be considered to bring about novel points of view that are functional to how new organism-world relationships are built and modified through experience; at the same time, they stimulate further creative behaviors as these formed relationships cannot persist if static. Instead, as recently argued by Schiavio and Benedek (2020), they constantly necessitate adjustments and modifications to remain interesting and open to the online wordly dynamics that take part in constituting one's musical identity.

In Western musical contexts, for example, we may consider how, despite much of the music we play is composed by others or develops from a shared repertoire of genre-specific gestures and forms, originality and innovation is nonetheless valorised and praised in musical interpretation. This may require a “dialogue” with the many performers who have recorded, shared, and discussed the music, where novel musical meanings can be formed and established. Moreover, in cases where original music is being created, composing musicians also tend to establish relationships—both actual and imagined—with past composers, performers, and audiences. This was recently investigated in a qualitative study with contemporary composers (Schiavio et al., 2022c), where it was found that the creative processes involved in music composition are often grounded in bodily experience and environmental, social exploration, emerging from the recursive interplay between composers and their social, cultural, historical, and physical niche. Along these lines, it has been argued that “[t]he most important aspects of musical creativity occur outside the head of the musicians” (Sawyer, 2006, p. 239). In a similar vein, the conceptual analysis offered in the present contribution suggests that minimal forms of the creative relationships can be found in entrainment, as we saw with infants' musical behaviors. Future work may further investigate the role of entrainment also in a variety of musical contexts typical of adult life, extending the preliminary insights offered in this paper. For example, it may be tested whether musical creativity is enhanced after taking part in coordinated activities with others, broadening the results of previous studies (e.g., Stupacher et al., 2017c) focused on the social consequences of synchronous behavior.

## Data availability statement

The original contributions presented in the study are included in the article/supplementary material, further inquiries can be directed to the corresponding author.

## Author contributions

AS: Writing – original draft, Writing – review & editing. MW: Writing – original draft, Writing – review & editing. JS: Writing – original draft, Writing – review & editing.

## Footnotes

^1^In other musical contexts, such as music production for instance, there is perhaps clearer mutual interaction between subjects and objects: musicians might choose a musical tempo that fits their breathing or swaying rate or be inspired by their interaction with the instrument to change existing musical plans on the spot (see Cook, 2013, 2018).

^2^The capacity to entrain and synchronize has been also documented in non-human animals (Fitch, 2013; Wilson and Cook, 2016).

^3^Polyrhythms consist of at least two different regular pulses that have no common root, such as 3:4.

^4^In light of the similarities between creativity and mental life described above, the claim might be extended to argue that entrainment is fundamental to the flourishing of mental life in itself. While this is certainly a fascinating insight, its thorough examination goes well beyond the aims of the present contribution.

^5^The scientific investigation of creativity centres on two closely linked concerns: establishing the concept of “creativity” in *products* and comprehending the cognitive and neural *processes* underlying it (see Fink et al., 2009; Abraham, 2018; Benedek et al., 2019). Characterizing creative cognition in scientific terms also requires considering both individual and collective factors (Schiavio and Benedek, 2020). Individual creativity involves divergent thinking and problem-solving (Guilford, 1950; Mednick, 1968), while research on collective creativity highlights communicative and participatory aspects (Amabile, 1996; Sawyer and De Zutter, 2009). Creative cognition does not necessarily have to be associated with an either/or dichotomy separating individual and collective forms of creativity. On the contrary, at the heart of creative thought and action, both endogenous and exogenous factors are often intertwined in a process of mutual specification, in which new meanings are discovered and contextually transformed through patterns of dynamic organism-world interactivity (Oyama et al., 2001). The relationship between individual and collective perspectives on creativity remains a topic of interest (Von Held, 2012).

^6^When a novel experience or sensorimotor association does not end up being “functional” enough to keep the entraining behavior interesting, the infant will likely try to discover novel motor possibilities (e.g., by moving other joints, modifying existing behavioral patterns, etc.), or terminate the body-world relationship established (i.e., by focusing on something else instead of the music).

^7^Here readers may be interested in drawing a comparison between these two terms and the concepts of *assimilation* and *accommodation* at the heart Piaget's genetic epistemology. In Piaget's approach, these concepts centre on how individuals incorporate new information into existing cognitive structures to construct new knowledge, while we use the notions of *adaptation* and *exploration* to emphasise the dynamic, action-based, and interactive nature of cognitive processes, extending beyond mere mental representation.
